# Why Evolutionary Psychology Should Abandon Modularity

**DOI:** 10.1177/1745691621997113

**Published:** 2021-11-03

**Authors:** David Pietraszewski, Annie E. Wertz

**Affiliations:** 1 Center for Adaptive Rationality, Max Planck Institute for Human Development, Berlin, Germany; 2 Max Planck Research Group Naturalistic Social Cognition, Max Planck Institute for Human Development, Berlin, Germany

**Keywords:** cognition, evolutionary psychology, Fodorian modularity, levels of analysis, modularity, philosophy

## Abstract

A debate surrounding modularity—the notion that the mind may be exclusively composed of distinct systems or modules—has held philosophers and psychologists captive for nearly 40 years. Concern about this thesis—which has come to be known as the *massive modularity debate—*serves as the primary grounds for skepticism of evolutionary psychology’s claims about the mind. In this article we argue that the entirety of this debate, and the very notion of massive modularity itself, is ill-posed and confused. In particular, it is based on a confusion about the level of analysis (or reduction) at which one is approaching the mind. Here we provide a framework for clarifying at what level of analysis one is approaching the mind and explain how a systemic failure to distinguish between different levels of analysis has led to profound misunderstandings of not only evolutionary psychology but also of the entire cognitivist enterprise of approaching the mind at the level of the mechanism. We furthermore suggest that confusions between different levels of analysis are endemic throughout the psychological sciences—extending well beyond issues of modularity and evolutionary psychology. Therefore, researchers in all areas should take preventive measures to avoid this confusion in the future.

Although most psychologists and philosophers of mind would grant some degree of modularity to the mind, evolutionary psychology in particular is considered extreme in its endorsement of *massive modularity*—the notion that the mind is mostly, if not entirely, composed of different systems or modules (Bechtel, 2003; Samuels, 2000, 2012). Massive modularity serves as a perennial grounds for skepticism or outright rejection of evolutionary psychology (for a review, see Goldfinch, 2015), and a lengthy debate on whether the mind is massively modular—or even in principle could be—has raged for more than 40 years with no resolution in sight (e.g., Barrett, 2005, 2007, 2015; Barrett et al., 2006; Barrett & Kurzban, 2006, 2012; Bechtel, 2003; Carruthers, 2003, 2005, 2006, 2007; Chiappe & Gardner, 2012; DeSteno et al., 2002; Ermer et al., 2007; Fodor, 1983, 1985, 1998, 2000; Frankenhuis & Ploeger, 2007; Goldfinch, 2015; Hagen, 2016; MacDonald, 2008; Newcombe et al., 2009; Samuels, 2000, 2012; Seok, 2006; Sperber, 1994, 2001; Tooby et al., 2005).

Oddly, given the length of this debate, there is not only disagreement about the degree of modularity within the mind but also about what is even meant by modularity. For at least 3 decades, successive waves of ink have been spilled in an attempt to clarify that evolutionary psychology does not subscribe to Fodor’s well-known criteria for modules (e.g., encapsulation and automaticity; Fodor, 1983). Rather, evolutionary psychology uses modularity simply to mean *functional specialization—*that is, that the mind is not an undifferentiated mass of equipotential associationist connections but is instead composed of heterogenous functions (Barrett, 2015; Barrett & Kurzban, 2006; Ermer et al., 2007). The response to this clarification has been to (a) ignore it (for a review, see Goldfinch, 2015), (b) suggest that this is not what evolutionary psychology meant in the past and that evolutionary psychology is now making a banal, shift-the-goalpost claim that applies to everything (Chiappe & Gardner, 2012; Morin, 2016), or (c) stipulate that functional specialization applies only to certain kinds of cognitive processes (such as peripheral, System 1 processes) but not to others (such as central, System 2 processes; Chiappe & Gardner, 2012; Fodor, 1983, 2000). In turn, the counterresponse from evolutionary psychology has been to (a) assert that functional specialization has been what evolutionary psychology meant all along (Barrett et al., 2006; Tooby et al., 2005), (b) question why dual-systems theory (i.e., System 1 and System 2) and Fodorian modularity seem to be interwoven concepts for these critics (Barrett, 2015), and (c) argue that functional specialization can apply to processes that land on either side of the distinctions made by both Fodor or dual-systems theory (Barrett, 2015; Barrett & Kurzban, 2012; Carruthers, 2003).

## Who’s On First?

The upshot of all this back-and-forth is that both sides in this debate believe that the other side is patently absurd in its convictions. Evolutionary psychologists cannot imagine what else could exist but functional specialization in the mind. Meanwhile, critics on the other side believe that the bottom has been pulled out from their understanding of evolutionary psychology if it does not intend the attributes of modularity that it now seems to be backing away from. Both sides are left, understandably, exasperated and at a seeming impasse.

The cost of this state of affairs cannot be overstated. It has misled an entire generation of scientists about how to think about the relationship between evolution and the mind, and it actively hinders progress in understanding how the mind works. However, this crisis represents a unique and powerful opportunity for clarification: Chronic intransigence typically indicates that there is not really a debate at all but rather a profound misunderstanding. Indeed, when reading through the enormous literature comprising the modularity debate it becomes increasingly clear that one is looking at a giant—but patently unfunny and scientifically tragic—version of Abbott and Costello’s “Who’s on First?” comedy routine (for video link, see NYYGehrig, 2012). For anyone unfamiliar with this routine, Costello attempts to ask Abbott about the names of players on a baseball team. However, the players all have names like Who, What, I Don’t Know, etc., which are then misinterpreted as evasive answers to Costello’s questions about the players’ identities. So when Costello asks, “Who’s on first?” Abbott replies, “Yes,” leading to an extended argument based on repeated misunderstandings of the meaning of the players’ names (and one of the classic comedy routines of the 20th century). Although Abbott and Costello never resolve their misunderstanding, the modularity debate is not doomed to the same fate. Instead, the vicious cycle of misunderstanding can be brought to an end by clearly articulating what both sides are arguing—which turns out to be decidedly different from what either side has believed the other to be saying up until now.

Our goal in this article is to dismantle the modularity debate entirely and show that it rests on a “Who’s on First?”–style misunderstanding—what we refer to as *the modularity mistake*. The modularity mistake can be succinctly summarized as follows. The debate until now has appeared as if two sides are quarreling about the extent of modularity within the mind and about what criteria should be assigned to modules. That is, it seems as if two sides are referring to the same entity—a *module*—but disagreeing about where it is and what it is like. But this is not what is happening. Rather, the two sides are simply talking past one another because each side is approaching the mind at a fundamentally different level of description or, as we prefer—and following Marr (1982)—a different *level of analysis*.

A level of analysis is a level of reduction or a level of explanation. In philosophical terms, each level constitutes its own ontology: a set of entities and rules stipulating how those entities can interact. As has been pointed out perennially by philosophers and scientists (e.g., Aristotle, ca. 350 B.C.E./1994; Dennett, 1987; Lorenz, 1948/1996; Marr, 1982), a full understanding of any complex entity or phenomenon requires description at more than one level. Moreover, each level of analysis complements the others. Different levels are not in direct competition with each other, and a description at one level does not obviate the need for a description at another. However, care must be taken to not confuse different levels of analysis because each level constitutes its own closed system. For this reason, unknowingly operating at different levels of analysis can create significant problems.

Take, for example, vanilla ice cream. One can describe (a) the way vanilla ice cream tastes when you eat it, (b) the structure of the vanillin molecule that is responsible for the vanilla taste, and (c) the physics of the particles that constitute the vanillin molecule. All three levels or descriptions—the taste, chemistry, and physics—are all valid scientific ways of describing the ice cream. Each constitutes what philosophers would refer to as a distinct ontology, meaning that each is its own independent causal framework, featuring a different set of entities and rules governing how those entities can interact. Thus, it is a category mistake (i.e., a confusion about what kind of thing something is) to combine levels or to think that the entities residing at one level can causally interact with entities residing at another. For example, it would be a mistake to think that the taste of vanilla can causally interact with a vanillin molecule or to think that a drawing of a vanillin molecule is somehow incomplete because the vanilla taste is not depicted somewhere within it.

Here, we argue that the levels of analysis framework is essential for understanding the debate surrounding modularity and why it has lasted for so long. Building on the previous ontologies of Dennett and Marr (e.g., Dennett, 1987; Marr, 1982), we present three levels of analysis—*intentional*, *functional*, and *implementational*. These three different ways of thinking about the mind each represent a different level of reduction, and each has its own validity. We argue that Fodor was operating mainly at the intentional level of analysis, whereas evolutionary psychology operates at the functional level of analysis. Neither side’s formulation of modularity makes sense within the other side’s level of analysis, and we show how much, if not all, of the controversy surrounding modularity is simply a consequence of each side operating at these different levels. We furthermore suggest that the unqualified concept of modularity be abandoned—not only by evolutionary psychologists but also whenever the term is applied to the mind—and be replaced with terminology that clearly denotes the level of analysis at which one is approaching the mind.

Our goal goes beyond simply articulating how damaging the “Who’s on First?”–style modularity mistake has been for evolutionary psychology or demonstrating how our new framework resolves these issues. Rather, we use the modularity mistake as an illustrative case study of what can happen when different level of analysis are confused with one another. This most basic of starting points—being clear about the level at which one is describing the mind—has been given remarkably short shrift by psychologists and philosophers of mind. This state of affairs is all the more troubling once one begins to notice that different levels of analysis are confounded frequently, and often without awareness, throughout the behavioral and psychological sciences. Explaining the modularity mistake is therefore the first step of a broader movement to resolve confusions stemming from unmarked shifts in perspective within psychology and philosophy of mind. If successful, this enterprise can tidy up nearly every area of inquiry in which humans seek to study themselves by clarifying at what level of analysis one is (and is not) operating within when approaching the mind.

### Levels of Analysis

If one hopes to achieve a full understanding of a system as complicated as a nervous system, a developing embryo, a set of metabolic pathways, a bottle of gas, or even a large computer program, then one must be prepared to contemplate different kinds of explanation at different levels of description.

—Marr, 1982 (p. 20)

**Fig. 1.**
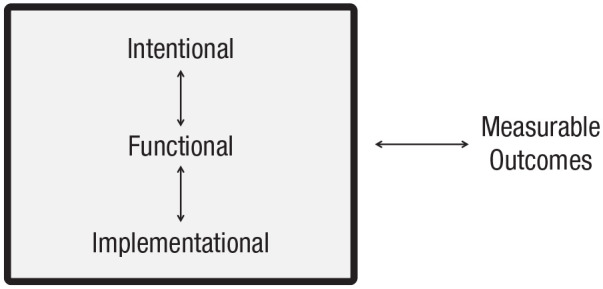
The causal processes within the mind responsible for producing measurable outcomes (such as observable behaviors or empirical data) can be understood at three different levels of analysis. These measurable outcomes afford inferences about the structure or content of the mind at any one of these three levels.

One of the great achievements of 20th-century psychology was the establishment of the kinds of descriptions and explanations that will be necessary for a complete science of the mind. There are two main components of this framework. First, a complete science of the mind cannot just describe measurable outcomes (i.e., experimental effects)—it must also appeal to latent variables (psychological entities) within the organism (e.g., Chomsky, 1959; Fodor, 1987a; Kendler, 1987; Tolman, 1925). Second, these psychological entities can be described at three different levels of reduction or analysis (Dennett, 1987, 1991a; see Fig. 1). Each level hosts different kinds of entities and rules of causation (i.e., each has a different ontology), and each sits at a different level of reduction than the others.

#### Intentional level of analysis

The *intentional* level of analysis is the least reductive of the levels and the default ontology that humans adopt when thinking about the mind. Elements of this level are studied under various names, including *theory of mind* and *psychological essentialism*, and the entirety of this level corresponds to what Dennett (1987) has called taking the *intentional stance*. At this level, a unitary *agency* or a “you” (i.e., an internal impetus; Starmans & Bloom, 2011) resides within the mind. This is the “self,” the “you,” or the “I” that peers out from the privileged location inside of our skulls, thinking, feeling, and making decisions. This agency—which we refer to here as the *central agency—*is sometimes thought of as a little person living inside of one’s head (a *homunculus*) or as a place where various operations of the mind come together to be evaluated and acted on by some holistic decision-making entity. This is the part of the mind where “you” are, where information has to arrive before “you” see it, and where “you” are in control (what Dennett [1991a] has called the *Cartesian theater*). This level also contains the first-person phenomenology of how things feel (e.g., being easy or effortful, under volitional control or automatic) and includes mental states such as emotions, beliefs, thoughts, desires, and so on (see Russell, 1921/2013).

Although this level can be scientific, it is intrinsically dualistic, meaning that some entities that fall within the body envelope are not seen as a part of the central agency but rather as separate from it (Starmans & Bloom, 2011). For example, one can lose one’s arm and not lose one’s self. The same applies to mental entities. For example, if I cannot retrieve a memory, that memory (at least for the time being) resides outside of my purview and becomes something separate from me. Thus, at this level, there is an agent who *directs* their attention, who *consults* their memories, and who *uses* strategies. These mental entities (e.g., attention, memories, strategies) are separate from the agent but are used by it (or interface with it). That is, in philosophical parlance, *interactionism* (Radner, 1971) is an essential feature of this ontology. Causation at this level involves an impetus that either originates from the agent (i.e., “I” meant to do it) or from any of the entities residing outside of it. These entities may be something in the external world or something internal but not within the purview of the homunculus (e.g., a reflex, or mechanisms that do not share my goal of weight loss and therefore tempt me with chocolate cake). A description of visual processes at this level would be, for example, that “I see objects in the world automatically but attend to different parts of a scene effortfully.”

#### Functional level of analysis

The next level down—the *functional* level—is mechanistic (Bechtel, 2008), which means that there is no longer any agent present or impetus involved. Rather, the entities that exist at this level, *mechanisms*, work by executing functions that are abstract input/output, or if/then, contingencies and rules of causation (Fodor, 1976, p. 83). This level corresponds to what Dennett (1987) has called the *design stance* and encompasses both of Marr’s *computational* and *algorithmic* and *representational* levels.^1^ Causation at this level occurs because of the particular constellation of functions being carried out across different mechanisms and the abstract if/then causal relationships between mechanisms (it is at this level that input/output equivalence exists; Dennett, 1995^2^).

At this level, there is no “you” or “I”; there is no “central” location where the operations of the mind come together—nor any exogenous agent or impetus sitting outside of any mechanism and acting on its outputs. Instead, only mechanisms exist. A description of vision at this level would feature descriptions of mechanistic input/output steps and the representations necessary for parsing objects in the world, including the abstract computational logic of color, size, and shape constancy, scene analysis, and so on.

Likewise, the intentional level description above that “I see objects in the world automatically but attend to different parts of a scene effortfully” would at this level be described exclusively in terms of the operation of mechanisms, whose functions in aggregate produce the intentional level description. For example, “I see” corresponds to the activation of a complex array of mechanistic functions—systems for representing lines, colors, occlusions, depths, and objects; classifying objects; communicating to conspecifics; and so on—none of which in themselves “see” and none of which ever becomes or interfaces with a unitary “I,” as the unitary “I” is itself another complex array of mechanistic functions at this level.

#### Implementational level of analysis

The third and most reductive level is the *implementational* level (Marr, 1982). This level describes the interactions between entities defined by their physical locations and attributes (e.g., the anatomical features and electrochemical processes of different brain regions) and corresponds to what Dennett (1987) has called the “physical stance.” Causation at this level occurs through the unfolding of physical events. A description of vision at this level would articulate how visual processes are physically instantiated. For example, electromagnetic radiation hits rhodopsin molecules housed within photoreceptors, leading to the electrochemical excitation of particular kinds of cells in the visual cortex, and so on (eventually all the way down to descriptions of the chemistry and physics of these steps).

#### The three levels

These three levels exhaust all known levels of description for the mind (see Adolphs, 2015), and we already intuitively appeal to these different levels when we think about ourselves from the neck down. For example, if you go to the doctor complaining of pain (an intentional-level description), you expect to hear about what system is malfunctioning (a functional-level description) and may be prescribed some kind of drug or surgery (to provide an implementation-level intervention). In other words, you might complain of searing pain in your back, your doctor would then explain this is caused by the fact that a nerve cell whose function is to relay pain signals is currently being physically pinched, which causes it to (mis)fire, and you may be prescribed an anti-inflammatory to reduce the swelling around the nerve. As this example demonstrates, (a) all three levels are complementary (one does not have to choose between feeling pain or having a pinched nerve), (b) the higher level gives meaning or significance to the next lower level, and (c) all three levels are important for a complete medical (or, in our case, scientific) account of what is happening.

### The Modularity Mistake

Although there is no alternative to using some combination of these three levels of analysis to describe the mind, there has not yet been adequate attention paid toward making the level at which one is operating explicit or to avoid cross-contamination (particularly between intentional and functional levels). Consequently, the adoption of these levels in psychology and philosophy of mind has been largely implicit, piecemeal, and confused. The functional level of analysis seems to be the most fragile and the least likely to be adopted. Indeed, in our experience, an appreciable number of behavioral scientists fail to recognize it entirely. This combination of factors has caused endless confusion about the claims different research traditions are making about the modularity of mind.

**Fig. 2.**
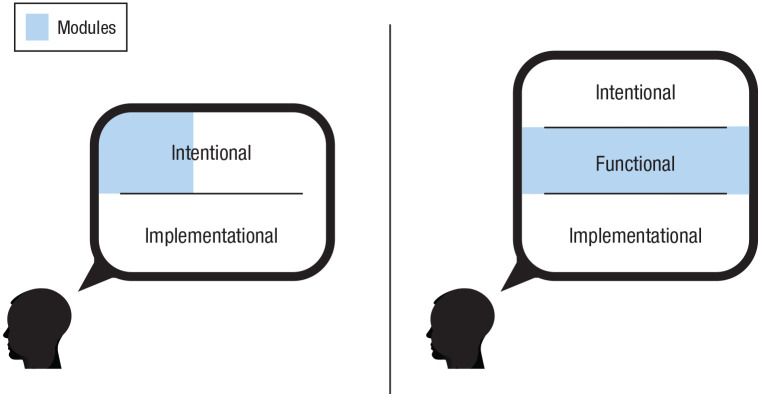
A visual depiction of the modularity mistake. One side of the debate, Fodorian modularity (on the left), conceives of modules as those entities that reside outside of the purview of a central agency—which is coherent at an intentional level of analysis. The other side of the debate, evolutionary psychology (on the right), conceives of modules as descriptions of the mind at a functional level of analysis. Using the same word, “module,” has sowed profound confusion in the back-and-forth debates surrounding modularity. In particular, criticism of evolutionary psychology’s claims of “massive modularity” in almost all cases stems from perceiving those claims through the lens of Fodorian modularity, which is simply incorrect.

Notably, Fodor’s articulation of modularity exists at an intentional level of analysis. As we articulate in detail below, the criteria that Fodor considered most important for modularity are only coherent at this level.^3^ Although he never explicitly summarized it in this way, Fodorian modules are the subset of entities in the mind that fall outside of the purview of a central agency. In contrast, evolutionary psychology’s notion of modularity—including the “updated” notion of functional specialization—is a discussion of entities falling entirely within the functional level of analysis (see Fig. 2).

Although Fodor had good reasons for articulating his conception of modularity at the intentional level, his use of this level was entirely implicit. Therefore, when evolutionary psychologists began to adopt the language of modularity (as a way of talking about the functional level of analysis; e.g., Cosmides & Tooby, 1992; Tooby & Cosmides, 1992^4^), Fodorian-informed philosophers and psychologists incorrectly interpreted this language through the lens of the intentional level of analysis.

This confusion of levels of analysis—what we call the *modularity mistake*—unleashed a cascade of profound misunderstandings that has wreaked havoc for decades. It has led to a perverse view of what evolutionary psychology is and what it is trying to do and, even more broadly, a perverse view of what is entailed by claims (coming from any theoretical perspective) that something is a function or a mechanism within the mind. Consequently, being explicit about the different levels of analysis at which claims or theoretical arguments are made will be vital not only for dispensing with the modularity mistake and an accurate view of the goals and worldview of evolutionary psychology but also for the long-term viability of the entire interdisciplinary cognitivist enterprise. No less is at stake.

### Fodorian Modularity and the Intentional Level of Analysis

To understand why Fodorian modularity exists at the intentional level of analysis, it is important to recognize where Fodorian modularity came from and why it was created. Fodor’s conception did not arise out of a vacuum. The implicit notion of modularity (in the explicit form of a “faculty”) has played an important role in physiology and medicine since at least Galen’s writings in the second century (Galen, 1523/1916) and later in psychology—as pointed out by Fodor^5^—beginning with Gall in the 19th century. The explicit concept of modularity began to appear in developmental and evolutionary biology around the 1930s (Needham, 1933), and by the 1960s it began to appear in artificial intelligence and software engineering (Simon, 1962, 1969/1996). We return to this original conception of modularity (which we argue is perfectly coherent) later, but for our purposes here it is sufficient to say that it had relatively little impact on psychologists’ and philosophers’ conceptualizations of the mind (Carruthers, 2003; although there were exceptions, e.g., Minsky, 1974/1975).

This would all change with the publication of Fodor’s (1983) book *The Modularity of Mind*. Fodor’s book arrived at a critical inflection point in psychology and philosophy of mind: Debates over behaviorism had recently run their course, and there was rekindled openness to the idea that the mind would be composed of a large number of diverse processes (H. Gardner, 1985; Minsky, 1974/1975, 1986). Researchers were settling into the enterprise of identifying what these processes might be and establishing how to talk about them (Kendler, 1987; Marr, 1982; Miller, 2003). Within this scientific context, Fodor proposed that some aspects of cognition have the attribute of being *modular*. In point of fact, however, much of the book is not about modularity but rather about “input systems” (see Box 1). Fodor’s analysis of input systems is beautiful, and there remains an active debate about their nature (e.g., Firestone & Scholl, 2016).

**Box 1**

**Modularity of Mind**

Anyone who comes to read *Modularity of Mind* by way of the modularity debate is likely to be surprised at just how little of the book is actually about modularity. Instead, the vast majority of the book (the first two thirds) is concerned with characterizing “input systems.”

Input systems sit downstream from sensory transducers (those things that transduce electromagnetic or mechanical energy into nerve conductance, such as on the cochlea or retina) and convey distal stuff out in the world into a format that can be used by the mind. Moreover,

since, in the general case, transducer outputs underdetermine perceptual analysis . . . we can think of each input system as a computational mechanism which projects and confirms a certain class of hypotheses on the basis of a certain body of data. (Fodor, 1983, p. 68)

Critically for Fodor, these hypotheses (guesses within the system about what is being seen or said) are drawn from “considerably less than the organism may know. That is, the confirmation function for input systems does not have access to all of the information that the organism internally represents” (p. 69). This is for a very good reason: Input analysis should be independent of “what the perceiver presumes or desires . . . at least for a fallible organism . . . it generally sees what’s there, not what it wants or expects to be there. Organisms that don’t do so become extinct” (p. 68).

Fodor refers to this “cordoning off” from the organism as “information encapsulation” (pp. 41, 67, 69, 71–73, 77, 80, etc.).

What is most important for our purposes here is that Fodor used the attributes of input systems as a vehicle to argue for a property—modularity*—*within the mind. In his words, “input systems constitute a natural kind” (Fodor, 1983, p. 99). For Fodor, input systems are by their nature informationally encapsulated, and it is this property that picks out a natural kind: modules. As he put it: “The key to modularity is information encapsulation” (p. 98). An entity is encapsulated if it “does not have access to all of the information that the organism internally represents” (p. 69; see also Box 1). Encapsulation is “the heart” (Fodor, 2000, p. 63) and “the essence” (Fodor, 1983, p. 71) of modularity. To fully understand what a Fodorian module is, it is also instructive to know what a Fodorian module is not. Fodor contrasted modules with *central systems* (see Box 2). These systems, like modules, are defined with respect to encapsulation. Unlike modules, however, central systems are not encapsulated—which means that they have full access to the organism’s beliefs, desires, and goals.

**Box 2**

**“Central” Systems**

As Fodor pointed out, “Mechanisms that operate as modules presuppose mechanisms that don’t” (Fodor, 2005, p. 71). He called these nonmodular, unencapsulated mechanisms *central systems*:

I assume that there must be relatively nondenominational (i.e., domain-inspecific) psychological systems which operate, inter alia, to exploit the information that input systems provide. Following the tradition, I shall call these “central” systems, and I will assume that it is the operation of these sorts of systems that people have in mind when they talk, pretheoretically, of such mental processes as thought and problem-solving. (Fodor, 2000, p. 103)

For Fodor, what is critical to these central systems is that they are for “belief fixation” (Fodor, 2000, pp. 112, 115, etc.), and that they have the attributes of being *Quineian* (“sensitive to properties of the entire belief system”; p. 107) and *isotropic*, meaning that propositions (beliefs, mental representations, etc.) are equally accessible—that is, “facts relevant” to a hypothesis “may be drawn from anywhere in the field” (p. 105).

Why did Fodor carve up the mind in this way? Scholars have suggested that Fodor was arguing against a prevailing assumption at the time of his writing that there would be some uniform “grand design” to all mental phenomena (Callebaut, 2005; Sperber, 1994). In other words, Fodorian modularity was an argument against content-blind, domain-general faculties (H. Gardner, 1985), or what Minsky (1974/1975) called *logistic* architectures, in which propositions embodying knowledge are separable from the rules of inference operating on them (Bechtel, 2003^6^).

Fodor’s modules codified a set of phenomena in which propositions are in fact cordoned off from the rest of the cognitive architecture. For example, in visual illusions, relevant explicit knowledge about what one is seeing cannot affect the outputs of visual perception (Fodor, 1983; Pinker, 2005; Rozin, 1976; Scholl & Leslie, 1999). Fodor’s modules thus served as an existence proof that the notion of a uniform, grand design could not be correct. Essentially, Fodor was asking whether any portions of the mind are closed off from its free-floating propositions. It is those entities that are “restricted” from these propositions (Fodor, 2000, p. 63) that are modules, whereas those entities that are not restricted are central systems.

Although Fodor did not outright state at which level he was operating in his writing, there is very little room for interpretation on this matter. *Encapsulation* meant isolation from the organism’s background beliefs, desires, and goals. This description could have been perfectly coherent at a functional level of analysis: Mechanisms, defined according to their function, have a particular purview. Therefore, Fodor could have meant that the mechanisms that underwrite input systems do not take as inputs any of the outputs coming from the mechanisms underwriting beliefs, desires, or goals. This would be similar to the way that the mechanisms that represent lines on the retina do not take as inputs any of the outputs of mechanisms for representing skin temperature. Fodor’s definition could thus be functionally defined as the scope of the computational purview of a mechanism.

But this is not what Fodor meant. Fodor acknowledged this *would* be a possible way to understand encapsulation:

It is a point of definition that distinct functional components cannot interface everywhere on pain of their ceasing to be distinct. It is this consideration that flow-chart notation captures by drawing boxes around the processing systems it postulates. That only the inputs and outputs of functionally individuated systems can mediate their information exchanges is tautological. (Fodor, 1983, p. 87)

In other words, at a functional level, each mechanism is defined according to what class of things it takes as inputs. Consequently, each mechanism is tautologically encapsulated because it cannot have as inputs other things outside of the inputs that it uses to execute its function (simply by definition).

But Fodor was decidedly *against* this understanding of modularity:

There is a usage according to which anything that is or purports to be a *functionally individuated* cognitive mechanism—anything that would have its proprietary box in a psychologist’s information flow diagram—thereby counts as a module. . . . In contrast . . . I shall simply take it for granted that cognition is typically the interaction of many functionally individuated parts, and use the “modularity thesis” as the name of something more tendentious. (Fodor, 2000, pp. 56–57)

Later he stated that “confusions of modularity with functional individuation have embarrassed the cog. sci. literature for several decades now; it really is time to stop” (Fodor, 2005, p. 29). Fodor was painfully, abundantly clear on this point; modules are not equivalent to functionally individuated entities.

So if Fodor did not mean functionally individuated entities in his distinction between modules and central systems, what did he mean, and at what level was he operating?

#### Fodorian modularity exists at the intentional level

**Fig. 3.**
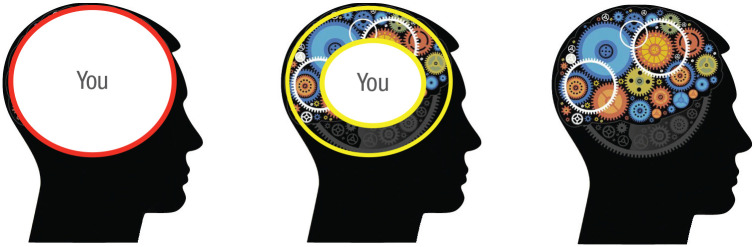
Three conceptions of the mind. Fodorian modularity is an argument against the view of the mind on the left—that “you” (i.e., a central agency) extends to cover all of the mind. It makes this argument by adopting the view in the center—that there are parts of the mind that do not include a central agency. However, at a functional level of analysis (the view on the right) even the phenomenological experience of a central agency—the “you”—is the result of a collection of mechanistic systems.

In fact, Fodor’s notion of informational encapsulation is meaningful only at the intentional level of analysis. This is because Fodor accepted the premise that a central agency exists in the form of central systems but then went on to argue that there are parts of the mind (i.e., modules) that do not fall within this region (see Fig. 3).

**Fig. 4.**
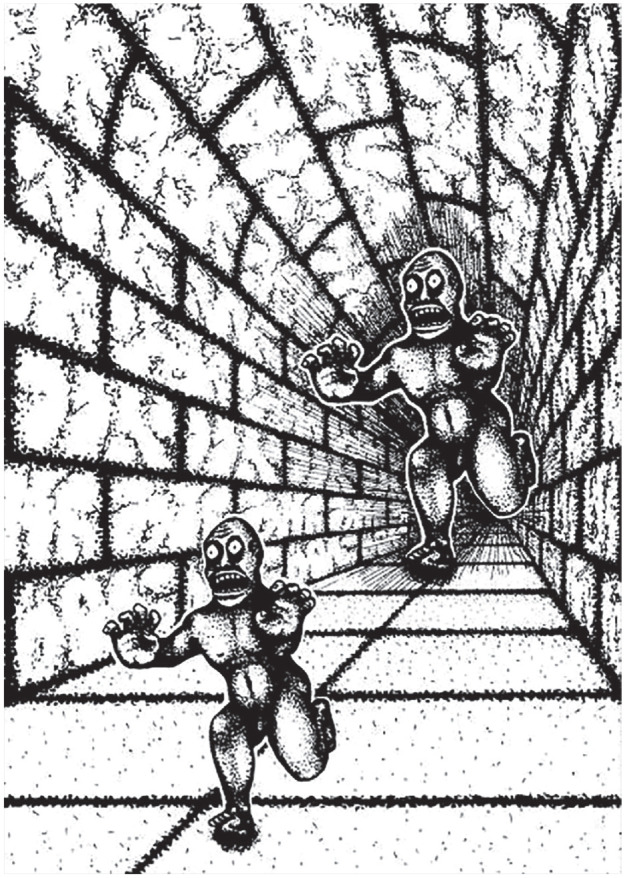
Roger Shepard’s *Terror Subterra* (copyright R. Shepard; reprinted with permission).

This fact can be demonstrated by examining a canonical example of modularity: visual illusions (Fodor, 1983; Pinker, 2005; Rozin, 1976; Scholl & Leslie, 1999). Figure 4 depicts a well-known visual illusion drawn by the cognitive scientist Roger Shepard. In this illusion, a large monster appears to be chasing a smaller monster down a corridor. However, the two monsters are in fact identically sized ink blotches; they subtend the same amount of visual angle on the retina. As is explained in introductory textbooks, the reason why the monsters are perceived as being different sizes has to do with an assumption of the visual system: In the real world, size and distance are conflated. Closer objects become optically larger, and objects farther away become smaller. To establish actual size, the visual system must take into account both the angle subtended on the retina and relative depth cues. In Shepard’s illusion, proximity to the vanishing point serves as a monocular depth cue. Therefore, the monster closer to the vanishing point appears farther away from the viewer. And because the two monsters subtend the same amount of visual angle on the retina, the one farther away is represented as being larger because in a real, three-dimensional scene it would be.

This illusion captures the point of Fodor’s modularity: One can learn from a textbook that the monsters are the same objective size and understand the reasons why, but that declarative knowledge cannot change the first-person experience of seeing a larger monster chasing a smaller one (or, as Fodor put it, “one simply cannot see the world under its retinal projection”; Fodor, 1983, p. 54). This phenomenon demonstrates that propositions are not uniformly accessible across the mind. Specifically, the proposition that the two monsters are the same size does not reach those parts of the mind responsible for producing conscious visual experience. Therefore, the mind is not purely isotropic (Fodor, 1983; Pinker, 2005; Rozin, 1976; Scholl & Leslie, 1999).

A skeptic might note that there is nothing yet in this example to indicate that Fodorian modularity exists at the intentional level of analysis. That is, the observation that content in one portion of the mind does not affect content in another is also perfectly compatible with adopting a functional level of analysis. But where the intentional level reveals itself is in Fodor’s treatment of encapsulation as being an *intrinsic* property of modules (as opposed to a *relational* property, as we discuss in detail below). Fodor used visual illusions to argue that visual input analyzers are modular because they have the property of being encapsulated. Recall that, for Fodor, something is encapsulated if it “has access, in the course of its computations, to less than all of the information at the disposal of the organism whose cognitive faculty it is” (Fodor, 1987a, p. 25); “encapsulated systems are prohibited by definition from considering all things” (Fodor, 1983, p. 89). That is, encapsulation makes sense only if there is a place where everything else comes together—what Fodor called central systems, which are “sensitive, at least in principle, to *everything* that the organism knows” (Fodor, 1983, p. 70).

Although such statements are perfectly coherent at an intentional level of analysis, they are perfectly incoherent at a functional level of analysis^6^: At a functional level of analysis, it is impossible for any mechanism to ever have access to all of the information at the disposal of the entire organism because the entire organism is itself composed of mechanisms—even processes that would fall under Fodor’s rubric of central systems (see Fig. 3). Therefore, it is not possible for there to be a place where all (or most) of the information will otherwise come together (see also Sperber, 2001). It is then meaningless to define a cognitive mechanism at the functional level according to whether it has access to all of the information at the disposal of the organism because this will never happen. It would be like defining a physiological process in the body—such as gas exchange across alveolar tissues in the lungs—according to whether that process had access to all of the other physiological processes occurring within the body. It is simply an incoherent statement.

Later we show how the notion of intrinsic encapsulation (or of intrinsic *separateness*) has wreaked havoc on the evidentiary standards held against researchers who adopt a functional level of analysis, such as evolutionary psychologists. For now, it is sufficient to note that appealing to encapsulation as an intrinsic property requires implicitly evoking a central agency from which an entity is isolated. No such thing exists at a functional level, but it does exist at an intentional level. In other words, the pitting of modules against central systems is simply a rebranding of the *me*/*not me* distinction within the intentional level of analysis. Something is intrinsically encapsulated (i.e., a module) when it resides outside of the purview of the central agency. Nonencapsulated central systems, in turn, *are* the central agency (for an explicit depiction of a Cartesian theater in a synopsis of Fodorian modularity, see, for instance, Coltheart, 1999, p. 116).

To be clear, we are not arguing that encapsulation (which is coherent at an intentional level of analysis) cannot have a corresponding functional level of analysis account. To return to an earlier example: Just as the taste of vanilla has a corresponding molecular description, so too does encapsulation have a corresponding functional-level description. In other words, the perfectly coherent intentional-level attribute encapsulated (or unencapsulated) is necessarily made possible by a set of cognitive mechanisms. Therefore, it has to be the case that each instance of an encapsulated (or unencapsulated) cognitive phenomenon can be described at a functional level of analysis. But what is important is that those mechanisms underwriting those phenomena do not themselves work by virtue of being encapsulated. That is, intrinsic encapsulation is meaningless at the functional level of analysis because all mechanisms at this level are all equally encapsulated: None is any more or less isolated from everything else in the mind.

In sum, then, encapsulation from the homunculus or Cartesian theater is perfectly coherent at an intentional level of analysis, but there is no coherent way to make Fodor’s attribute of encapsulation an intrinsic property of a mechanism at a functional level of analysis.^8^ Yet, to our knowledge, this simple fact has been missed by critics and proponents of Fodorian modularity alike.

Unfortunately, and even more confusingly from the perspective of trying to keep levels of analysis clear, Fodor included an additional set of attributes for modules (see Box 3). None did he deem as important as encapsulation (e.g., Fodor, 1983, pp. 71, 98, 110). Rather, he suggested that these attributes might co-occur with encapsulated modules in a fuzzy-set, family-resemblance kind of way (e.g., Fodor, 1983, pp. 99–101). The reason why these attributes create additional confusion about levels of analysis is that some are obviously at the intentional level of analysis (e.g., automaticity), whereas others appear as if they might exist at a functional level of analysis (e.g., domain specificity). For example, automaticity, like encapsulation, is treated as an intrinsic property of mechanisms (e.g., Fodor, 1983, 2000; Samuels, 2000, 2012) and thus is meaningless at a functional level of analysis. Automaticity, by definition, entails the lack of some agency that is present for nonautomatic processes (e.g., an automatic transmission in a car is the kind that does not need to be directly operated by the driver, unlike a manual transmission).^9^ In contrast, domain specificity—which describes a class of inputs to which a mechanism is sensitive (see Boyer & Barrett, 2016)—does not require a central agency to be coherent.

**Box 3**

**Additional Attributes of Fodor’s Modules**

In addition to information encapsulation, Fodor also—reluctantly at times—suggested that modules may tend to have the following other attributes, which may more or less stick together:

**Automatic**

“Because these processes are automatic, you save computation (hence time) that would otherwise have to be devoted to deciding whether, and how, they ought to be performed” (Fodor, 1983, p. 64).

**Fast**

“Eyeblink is a fast response because it is a reflex—i.e., because you don’t have to decide whether to blink your eye” (Fodor, 1983, p. 64; generally pp. 61–64).

**Domain-specific**

“The more eccentric a stimulus domain, the more plausible the speculation that is it computed by a special-purpose mechanism” (Fodor, 1983, p. 51).

**Fixed neural architecture**

“Hardwired connections indicate privileged paths of informational access; the effect of hard-wiring is thus to facilitate the flow of information from one neural structure to another” (Fodor, 1983, p. 98).

**Characteristic development**

“The neural mechanisms subserving input analysis develop according to specific, endogenously determined patterns under the impact of environmental releasers” (Fodor, 1983, p. 100).

**Shallow outputs**

Outputs are not elaborated on by “background knowledge” (Fodor, 1983, p. 87) but are not so shallow that they are not “phenomenologically accessible” (p. 88; see also pp. 93–94, 96).

**Characteristic breakdown**

“Input systems exhibit characteristic and specific breakdown patterns” (Fodor, 1983, p. 99).

Because this article is not a complete review of Fodorian modularity, we will not go any further into Fodor’s other modularity criteria, aside from noting that encapsulation and automaticity are widely considered the key, defining elements of Fodor’s concept of modularity (the latter primarily by others other than Fodor; e.g., Bechtel, 2003; Coltheart, 1999; Fodor, 1983, 1985, 1998, 2000; Frankenhuis & Ploeger, 2007; Samuels, 2000, 2012; Seok, 2006; Sperber, 1994). This is precisely because Fodor was arguing against a purely isotropic mind, and therefore the attribute of being cordoned off from what seems to be an otherwise open pool of propositions is central to Fodor’s argument.

But we would argue that Fodor’s true argument was something far more profound—an argument against the notion that “you” are in complete control of how your mind works. Modules are those things in the mind that are not “you.” This is why, according to Fodor’s criteria for identifying modules, modules are automatic or mandatory—because “you” cannot change them. This is why there is limited central access—because you cannot get to them. This is why there are shallow inputs—because deep inputs would get to “you,” and “you” would be able to elaborate and act on those inputs. And this is why modules are separated from central systems (i.e., “you”). To see Fodor’s criteria used in this manner, see, for instance, Coltheart (1999), Fodor (1983, 2000), and Samuels (2000, 2012).^10^

Indeed, although it has been pointed out elsewhere that Fodor’s notion of modularity is intrinsically dualistic—for example, Barrett (2015) referred to it as an “igloo model” of the mind—we would argue that this is a feature of Fodor’s approach, not a bug. Fodor’s conceptualization of modularity did not gain traction because of wide-ranging interest in issues of isotropy. Rather, Fodorian modularity became wildly popular because it confronted near-universal, everyday intuitions about how the mind works—that “you” are more or less in complete control of your mind. Fodor’s modules did the important work of retracting the boundaries of where “you” (the central agency) resides—and did so in a compelling way by addressing the issue at the intentional level of analysis, which is the way that people intuitively and naturally approach the mind. Indeed, Fodor himself seemed to acknowledge the continued existence of something like a central agency in his theoretical framework:

A lot is known about the transformations of representations which serve to get information into a form appropriate for central processing; practically nothing is known about what happens after the information gets there. The ghost has been chased further back into the machine, but it has not been exorcised. (Fodor, 2000, p. 127)

### Evolutionary Psychology and the Functional Level of Analysis

Cognitive processes, like electrons, are entities defined solely by input-output relations.

—Cosmides (1985, p. 2)

In contrast to Fodor’s framework for carving up processes in the mind, evolutionary psychology approaches the mind primarily at a functional level of analysis^11^ (e.g., Barrett, 2015; Buss, 1995; Conway & Schaller, 2002; Cosmides, 1985; Cosmides & Tooby, 1987, 1994a, 1994b; Daly & Wilson, 1986, 1988; Pinker, 1997; Smith & Winterhalder, 1992; Symons, 1979, 1987, 1992; Tooby & Cosmides, 1990, 1992, 2016). At this level of analysis, the entity making decisions is not a central agency but instead a constellation of mechanisms. Mechanisms are material things that execute some function, and the function is defined by the problem to be solved (e.g., holding open a door, digesting meat, vacuuming a room, or avoiding predators; see Cosmides & Tooby, 1987, 1994b; Dennett, 1995).

Within the functional level of analysis, mechanisms and their functions can be described at different *degrees of abstraction*. For example, the entire digestive system is in some sense a mechanism because it can be described as having the function of digesting food: taking in food and then performing digestive operations that eventually lead to the output of delivering bioavailable raw materials out of which the organism is built, maintained, and run. Moreover, the small intestine can equally be described as a mechanism—executing the more specific function of absorbing nutrients and minerals. So too can villi, specialized microfingers within the small intestine that capture particular classes of nutrients via diffusion. Villi are in turn composed of microvilli, and so on. Each description here meaningfully constitutes a mechanism because each description captures the execution of a particular function.

Consequently, there is no one scale at which “the mechanism” exists. There are as many mechanisms as there are ways of describing functions. Therefore, it is not terribly meaningful to ask how many mechanisms there are. Rather, it is more meaningful to ask what the functions are and to answer that question as precisely as possible. In an evolutionary framework, these bits of functional mechanism are called *adaptations* and are characterized or described in terms of their purpose and how they work (Buss, 1995, 2016; Cronk et al., 2000; Davies et al., 2012; Dennett, 1995; A. Gardner, 2009; Grafen, 2007; Lorenz, 1948/1996; Pinker, 1997; Smith & Winterhalder, 1992; Tooby & Cosmides, 1990, 1992, 2016; Williams, 1966; Winterhalder & Smith, 2000). What evolutionary psychologists have meant by the *functional specialization* of the mind, then, is that the mind is composed of many different mechanisms, each of which can described according to its function (e.g., Barrett, 2006, 2015; Barrett & Kurzban, 2012; Tooby et al., 2005).

Adopting this functional level of analysis—which of course is not unique to an evolutionary approach—becomes particularly critical for applying evolution to psychology and behavior (Buss, 1995; Cosmides & Tooby, 1987; Daly & Wilson, 1988; Symons, 1979). Intuitively, we often think of the whole person as the entity making decisions and that attributes of the person modify those decision-making proclivities: Some people are stingy, some generous, and so on. Evolution applied to behavior is often (incorrectly) thought of in terms of these kinds of traits. That is, it can be tempting to think that evolution—or more precisely, a history of evolution—modifies or exerts a pull on what the individual would otherwise choose or do. This way of thinking is often characterized by the language of “bias” or “predisposition.” However, this is not the correct way to think about evolution applied to behavior. Evolution cannot be a partial contribution to the person because all of the processes that make up the person—everything that allows the person to think, plan, feel, learn, decide, and so on—are the result of adaptations,^12^ by-products of those adaptations, or noise (Barrett, 2015; Buss et al., 1998; Neuberg et al., 2010; Tooby & Cosmides, 1990, 1992). Moreover, evolution cannot directly act on behavior. Thus, the link between evolution and behavior is found in the form and function of the mechanisms for producing behavior—the organism control systems typically studied under the rubric of psychology (Cosmides & Tooby, 1987; Daly & Wilson, 1986, 1988; Dennett, 1995; Smith & Winterhalder, 1992; Symons, 1979, 1987, 1992). In evolutionary biological terms, these control systems are called the proximate phenotype, or more specifically, the proximate psychology (see also Scott-Phillips et al., 2011).

One of the insights of the 20th century was that all information-processing devices, including the behavioral control systems within organisms, can be characterized as a set of mechanistic if/then contingency rules (Turing, 1950; see also Pietraszewski, 2020). Consequently, all of the psychological mechanisms for producing behavior can also be described as sets of nested if/then contingency rules. At each scale, such mechanisms or systems take particular classes of entities in as inputs, perform some operation or process on those inputs, and then generate some output. This input/output level of description is the functional level of analysis (Dennett, 1987) and mirrors exactly how one can describe how the rest of the body works (from cells to organs or to entire systems, such as the digestive system) in terms of each mechanism’s role or function (Block, 1998; Cosmides & Tooby, 1987; Dennett, 1995; Tooby & Cosmides, 1992).

**Fig. 5.**
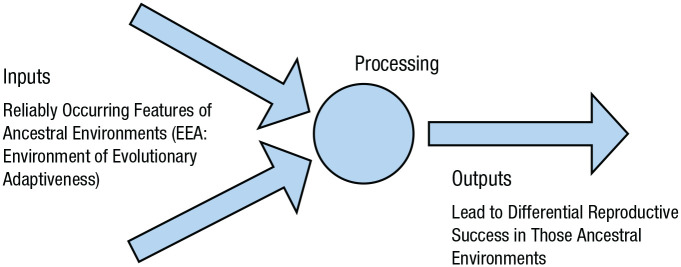
An axiom of adaptationism. All biologically evolved contingency systems within organisms, including the human brain, are constrained by natural selection to embody the following relationship: They take as inputs features of the environment that were reliably present over evolutionary time and generate outputs that would have been selected for over evolutionary time. All nonentropic phenomena produced by organisms are the result of some combination of such systems (adapted from Pietraszewski, 2020).

Evolutionary processes dictate the form of the if/then contingencies for each mechanism. Because natural selection is the only known force that creates biological complexity (A. Gardner, 2009), and because natural selection works in a particular way (Grafen, 2007), all if/then contingency rules are built according to the same fundamental logic: They will take as inputs those features of the environment that were reliably present over multiple generations of evolutionary time and generate outputs that would have been selected for within that environment (Buss, 1995; Cosmides & Tooby, 1987; Daly & Wilson, 1988; Tooby & Cosmides, 1992; see Fig. 5). The “environment” here refers to those features that are stable enough to have interacted with mechanisms over multiple generations, thereby shaping the structure of those mechanisms (Lewis et al., 2017; Symons, 1992; Tooby & Cosmides, 1990; Wertz & Moya, 2019). Despite frequent misconceptions, “learning” is not an exception to this principle. Rather, learning mechanisms are themselves a class of evolved if/then contingency rules and fall squarely within an evolutionary analysis (for some examples, see Barrett, 2015, 2016; Boyd & Richerson, 1985; Gallistel, 2000; Oña et al., 2019, Tooby & Cosmides, 1992, 2016; Wertz, 2019).

This conceptualization of mechanisms as evolved input/output devices, as depicted in Figure 5, is simply another way of restating the principles that (a) all mechanisms at a functional level of analysis can be described in terms of their inputs, processing, and outputs, and (b) all biologically evolved mechanisms described at this level are the product of natural selection, which constrains what these inputs, processing, and outputs can be (see also Buss, 1995). Properly understood, evolutionary psychology is then simply the wing of the evolutionary social sciences that is concerned with proposing and testing for what the input/process/output mechanisms in the mind might be (Barrett, 2015; Buss, 1995; Conway & Schaller, 2002; Cosmides & Tooby, 1987, 1994b; Lewis et al., 2017; Neuberg et al., 2010; Symons, 1992). In other words, it is an approach to the mind that marries the functional level of analysis to adaptationism (Tooby & Cosmides, 1992, 2016).

Two additional concepts are critical for understanding evolutionary psychology: *evolvability* and *computational adequacy*. Evolvability asks whether a postulated input/process/output mechanism could in principle have evolved (Andrews et al., 2002; Tooby & Cosmides, 1992). In particular, evolutionary psychologists constrain themselves to positing only biological mechanisms for dealing within inputs that would in principle have been recurrent over evolutionary time (and therefore cannot propose mechanisms that take as inputs evolutionary novelties, unless these inputs are taken in as a side effect or by-product of the mechanism’s evolved structure; e.g., Park, 2007; Tooby et al., 2003). This is why evolutionary psychologists are explicit in their assumptions about ancestral environments (Symons, 1992; Tooby & Cosmides, 1990; Winterhalder & Smith, 2000). Operating within the constraints of evolvability also means that evolutionary psychologists constrain themselves to positing mechanisms that generate outputs that would have been selected for on average over evolutionary time (what outputs would be selected for are in turn informed by optimality analyses from behavioral ecology and models of evolvability from evolutionary modeling and game theory; e.g., see Buss, 2016; Cronk et al., 2000; A. Gardner, 2009; Grafen, 2007; Smith & Winterhalder, 1992; Winterhalder & Smith, 2000). This is why evolutionary psychologists focus on *adaptive problems* (i.e., problems that have consequences for the long-term differential reproductive success of mechanisms; Tooby & Cosmides, 1992).

*Computational adequacy* refers to whether a specified mechanism can adequately solve a particular problem (Chomsky, 1980; Marr, 1982; Minsky, 1961, 1974/1975; Tooby & Cosmides, 1992). That is, if the description of the mechanism were to be implemented, would that implementation produce the same kinds of observables or behaviors that we see in the biological system, without the help of an intervening central agency or experimenter? To the degree the answer is “yes,” then the mechanism is computationally adequate.

The twin issues of evolvability and computational adequacy explain why evolutionary psychology has historically posited much more structure to the mind than many of its contemporaries. Adaptive problems are specific, real-world problems—such as finding food; finding a mate; staying alive and thriving in the face of resource exchanges, conflicts, and ever-shifting social alliances; and so on at every stage of the life span (for reviews, see Buss, 2016; Cronk et al., 2000; Davies et al., 2012). When one’s goal is to address these problems in a computationally adequate way one quickly realizes the inadequacy of logistic, content-neutral (i.e., “domain-general”) architectures, or positing that high-level abstractions such as “memory,” “attention,” or so on can adequately describe how these problems are solved. Indeed, whenever researchers have the long-term goal of completely solving the information-engineering problems of the real world, regardless of whether they take an evolutionary approach (such as in artificial intelligence; e.g., Minsky, 1961, 1974/1975, 2006; Mueller, 2015; Wall & Brock, 2019), one finds similar views regarding the number and heterogeneity of information-processing functions that must be accounted for.

The above concepts are essential to understanding what evolutionary psychologists have had in mind when they have appealed to the concept of modularity in the past. Consider the well-known cheater-detection work of Cosmides and colleagues (e.g., Cosmides, 1985, 1989; Cosmides & Tooby, 1989, 1992, 2015). The goal of the research program is not to show that cheater detection meets the criteria for a Fodorian module (Tooby et al., 2005) but rather to empirically examine whether the predicted design solutions for a particular information-processing problem—in this case, not being exploited in social exchanges—do in fact exist in the mind (for evidence collected thus far, see Cosmides, 1985, 1989; Cosmides et al., 2010; Cosmides & Tooby, 1989, 1992, 2015). To claim that a cheater-detection module exists in the mind is to claim that social exchange is (a) an evolutionarily recurrent problem with a clear set of selection pressures (i.e., it satisfies evolvability constraints) and (b) not being exploited in social exchanges requires specifying a set of information-processing functions within the mind that are adequate for solving this problem (i.e., the proposed solutions must be computationally adequate; for an in-depth task analysis of the information-processing requirements, see Cosmides & Tooby, 1989). The cheater-detection “module” is therefore defined according to its inputs and outputs: It takes as input those events in which an agent intentionally attempts to acquire a benefit without paying a cost or satisfying a requirement and then outputs the detection of those events to other systems involved in orchestrating antiexploitation responses (see Cosmides & Tooby, 2015).

In sum, evolutionary psychology operates at the functional level of analysis, and modularity has been evoked within evolutionary psychology to describe mechanisms defined according to their functions (i.e., functional specialization; Barrett & Kurzban, 2006, 2012). These functionally specified mechanisms can be described in the language of inputs and outputs and are constrained by the principles of evolvability and computational adequacy. The nature of each adaptive problem determines the attributes of the mechanism(s) that solves it—the form of the mechanism fits its function (Barrett, 2015). Moreover, there is no entailment that cheater detection, or any other proposed mechanism, should have the intentional-level attributes of being encapsulated or automatic, as would be the interpretation under Fodorian modularity (Tooby et al., 2005). These tenets have been central to evolutionary psychology since its inception and continue to be: “When thinking about the design features of mechanisms, we can think about the design of all three of these aspects—inputs, operations, and outputs—in adaptationist terms” (Barrett, 2015, p. 273). Evolutionary psychology makes no other axiomatic claims about the mind.

### Fodorian Modularity, Evolutionary Psychology, and the Modularity Mistake

Given that Fodorian modularity approaches the mind largely at an intentional level of analysis and evolutionary psychology approaches the mind largely at a functional level of analysis, how did the two become mixed up with one another? The modularity mistake emerged in part because of word choice. Both Fodor and evolutionary psychologists used the same word, “module,” to refer an entity at each of their respective levels of analysis. As Carruthers (2006) pointed out, Fodor had an outsized impact on philosophers of mind and psychologists, many of whom first encountered modularity by way of his book. Fodor’s book also arrived earlier than most of the foundational publications in evolutionary psychology. So, by the time evolutionary psychologists began to appeal to modularity (e.g., Cosmides & Tooby, 1997; Gallistel, 2000; Tooby & Cosmides, 1992), the concept was already widely understood through the lens of Fodor’s intentional level of analysis.^13^

However, word choice should not be given too much credit (or blame) for the confusion between the intentional and functional levels of analysis. An intentional level of analysis is the default way that people naturally think about the mind (Churchland, 1981; Dennett, 1987; Knobe, 2006; Starmans & Bloom, 2011), and this default is exceedingly difficult to overcome, even for scientists (Braitenberg, 1984; Cosmides & Tooby, 1994b). If Fodor had approached the mind at a functional level of analysis, he, too, would likely have struggled against an interpretation of his version of modularity through the lens of the intentional level of analysis, just as evolutionary psychology has.

Once one understands that the modularity mistake takes place amid a fundamental confusion between intentional and functional levels of analysis, the content of the modularity debate becomes predictable (almost uninterestingly so). A main point of contention is evolutionary psychology’s claim that the mind is composed entirely of modules rather than just containing a handful at the periphery—a thesis that came to be known as massive modularity (e.g., Bechtel, 2003; Samuels, 2000, 2012; Sperber, 1994, 2001). Massive modularity is then considered a radical position, sparking decades of debate (e.g., Chiappe & Gardner, 2012; DeSteno et al., 2002; Ermer et al., 2007; Fodor, 1998, 2000; Frankenhuis & Ploeger, 2007; Hagen, 2016; MacDonald, 2008; Newcombe et al., 2009; Samuels, 2000, 2012; Seok, 2006).

Of course, viewed within the correct functional level of analysis, evolutionary psychology’s claims of so-called massive modularity are not radical at all. If anything, they are boringly axiomatic. The claim is simply a logical entailment of Darwin’s theory of natural selection: If one is a materialist, then one must accept that organisms are composed of packets of evolved functions, their by-products, and noise (for an extended discussion, see Barrett, 2015; Buss et al., 1998; Dennett, 1995; Park, 2007; Williams, 1966). There is currently no known viable alternative. Therefore, the mind must also be composed entirely of modules—if by “modules” one means evolved functions (i.e., mechanisms)—by-products of their operation, and noise. Of course, there is plenty of room for debate and rancor over exactly what those evolved functions are and the degree to which particular outcomes reflect true biological adaptations rather than by-products or noise, but these were not the issues being debated. Instead, it was the very notion that the mind could in principle be massively modular that was treated as problematic (e.g., Chiappe & Gardner, 2012; DeSteno et al., 2002; Fodor, 1998, 2000; Goldfinch, 2015; MacDonald, 2008; Newcombe et al., 2009). In other words, evolutionary psychologists were happy to argue about which functional systems exist and how those systems may be structured, but they found it absurd that they must defend the very notion of modularity itself.

In contrast, from Fodor’s intentional level of analysis, a massively modular mind would be problematic. Within this framework, modules are inflexible, autonomous, and independent. Thus, a massively modular mind would be composed of largely isolated, inflexible units with limited ability to communicate with one another or with “central systems” (the homunculus or Cartesian theater). This kind of mind would be a many headed monster, and it would be exceedingly reasonable to question the plausibility (or even the logical coherence) of such a mental architecture.

Evolutionary psychologists *did* defend—and try to explain—their position to critics who misunderstood their approach. But at no point in time did evolutionary psychologists explicitly point out that they were adopting a different level of analysis than was Fodor. Instead, the debate centered around what attributes modules should have. As a result, the misunderstandings persisted.

For example, in what is probably the strongest and most recent attempt to clarify what evolutionary psychologists mean when they invoke modularity, Barrett and Kurzban (2006) explicitly rejected Fodor’s long list of attributes and instead offered (again) the simpler notion of functional specialization—“that mental phenomena arise from the operation of multiple distinct processes rather than a single undifferentiated one” (p. 628). This argument is exactly correct at a functional level of analysis and so would be the right argument to make if everyone was already clear about the level of analysis at which they were operating. However, because everyone was not already clear on this issue, critics of evolutionary psychology simply continued to interpret (and therefore misunderstand) this clarification through the lens of their intentional level of analysis (for an example, see Box 4).^14^

**Box 4**

**An Example of the Modularity Mistake**

The following back and forth between Chiappe and Gardner (2012) and Barrett and Kurzban (2012) exemplifies the confusion between different levels of analysis in the modularity debate. In the first quote, Chiappe and Gardner criticize the Barrett and Kurzban (2006) clarification that by “module” evolutionary psychology simply means a unit of functional specialization:

Barrett and Kurzban (2006) fail to adequately deal with the challenges posed by novelty. The reason is because they attempt to deal with it using only the System 1 processes traditionally discussed by EP. Specifically, they try to reduce the problem of novelty to one that can be dealt with by relying on Sperber’s (1994) distinction between the proper and actual domain of modules. The proper domain of a module is the set of inputs that a module evolved to process. The actual domain refers to stimuli that are similar in relevant respects to the proper domain of a module. . . . However, this does not eliminate the problem of novelty . . . one cannot always rely on the lucky coincidence where a novel stimulus just happens to fit the input criteria of a module, and whose operation is going to produce a suitable response to that stimulus. In other words, sometimes we have to deal with novelty by engaging in problem solving. Sometimes we actually have to think about a problem and gain insight into it so that we can improvise a solution. We can’t rely on a prepared response produced by natural selection. This can require considerable effort and ingenuity. (Chiappe & Gardner, 2012, p. 679)

Barrett and Kurzban responded as follows:

Our view is not that the System 1/System 2 distinction is necessarily useless, at least when defined in terms of “automaticity”: for example, some kinds of processes do appear to respond to subjects’ self-reported “volition” more than others (Wegner, 2002). However, we believe that it is a mistake to think of the “automatic” systems as being the result of evolution by natural selection, and the other systems as being the result of something else. . . . If this is right, then an evolutionary “modular” view is likely to illuminate both the functions and functional design features of System 2 processes. (Barrett & Kurzban, 2012, p. 685)

In the first quote, Chiappe and Gardner are operating at the intentional level of analysis: They suggest that mechanisms limited by their inputs are not flexible enough to deal with novelty, so rather a “one” or a “we” must “think” about the problem using “effort” and “ingenuity.” Of course, at a functional level of analysis, there only are mechanisms and their inputs. The ontology in which there exists mechanisms limited by their inputs on the one hand and a flexible agent (denoted by personal pronouns of “one” and the plural “we”) on the other is the intentional level of analysis. As a result, this is a clear instance of misunderstanding evolutionary psychology through the incorrect lens of the intentional level of analysis.

In the second quote, Barrett and Kurzban are operating at the functional level of analysis: They state that both sides of the me/not-me distinction at the intentional level of analysis have a corresponding functional-level description and that the entirety of that description is composed of evolved mechanisms. However, by charitably switching back and forth between levels (i.e., meeting Chiappe and Gardner halfway by suggesting that there are “automatic” systems and those that are not automatic), the difference in the level of analysis between the two sides remains obscure, even though what they are saying in this response is exactly correct.

In short, this is the heart of the modularity mistake: Both sides were arguing about the extent and meaning of modularity but all the while were referring to completely different levels of analysis. Such a debate has all the scientific merit of two people looking at a long, rectangular box from two different vantage points—one looking at the long side, the other at the short side—and then arguing about its dimensions.

### The Consequences of the Modularity Mistake

The modularity mistake has not only prolonged what is, essentially, an unnecessary debate over what is meant by (massive) modularity. It has also actively hindered progress into what we are all ostensibly interested in doing—figuring out how the mind works. In particular, the modularity mistake has led an appreciable number of researchers—possibly even the majority of behavioral scientists—to fundamentally misunderstand the goals and worldview of evolutionary psychology. In its most charitable form, this misunderstanding has caused researchers who share the same goals as evolutionary psychologists to believe instead that the approach is something else entirely, thereby robbing both sides of opportunities for mutually informative collaboration. In its most cynical form, this misunderstanding serves either as a misdirection away from the real issues of evolvability and computational adequacy by theories that lack either or a way to reassure oneself of one’s own theoretical sophistication by being “at least not as bad as those evolutionary psychologists.”

Although the harms caused by the modularity mistake specifically (let alone confusions surrounding different levels of analysis broadly) have been immeasurable, the following sections address what are to us the two broadest problematic consequences relevant specifically to evolutionary psychology. These consequences capture the heart of the modularity mistake’s impact and serve as a cautionary tale for other areas of the cognitive sciences in which confusing levels of analysis results in fundamental misunderstandings.

#### Misunderstanding evolutionary psychology through the lens of an intentional level of analysis

Through the incorrect intentional-level lens, evolutionary psychology has come to be seen by many researchers as an enterprise in which one is trying to demonstrate that something is an evolved mechanism by showing that it falls outside the purview of the central agency (e.g., DeSteno et al., 2002, 2006). In other words, from this viewpoint, the influence of evolution on the mind becomes (incorrectly) narrowed to just those processes that are inflexible, autonomous, and independent of “you.”

What follows logically from this flawed way of thinking is that researchers can (and should) empirically test for the influence of evolution on the mind by determining which mental phenomena fall outside of one’s control (i.e., seem “automatic” or “inflexible”). If the phenomenon is under “your” control, it is placed into the nonevolved, nonmodular bin. If instead the phenomenon is not under “your” control, then it is placed into the evolved, modular bin (e.g., see Chiappe & Gardner, 2012; DeSteno et al., 2002, 2006; MacDonald, 2008; see also Box 4).^15^ Any theoretical framing in which evolution is pitted against flexibility, rationality, or conscious deliberation is invariably an example of approaching claims about evolved cognitive processes through the lens of the intentional level of analysis (for many additional examples, see the work reviewed in Goldfinch, 2015).

Evolutionary psychologists have responded to this mistaken perspective and in the process have at times acted as if they accept its premise. That is, evolutionary psychologists have sometimes adopted the habit of explicitly trying to document effects that are automatic, unconscious, mandatory, and not intuitively rational (e.g., Haley & Fessler, 2005; cf. Dear et al., 2019; for a discussion, see Jung et al., 2012). Likewise, manuscripts informed by evolutionary theorizing are frequently rejected during the review process if the results can be described as originating from a central agency (e.g., reasoning, deliberative thought).

To be clear, there is nothing inherently mistaken about documenting cognitive processes that, from an intentional-level perspective, have attributes of automaticity, irrationality, and so on. The error is to assume, and to perpetuate the misunderstanding, that *only* those processes that bear these attributes can be considered the result of evolutionary processes. There is nothing in the theoretical framework of the evolutionary social sciences that says that adaptations have to be fast, encapsulated, and automatic or have limited central access and shallow outputs—even if we consider each one of these characteristics within their appropriate level of analysis. Natural selection does not obey Jerry Fodor’s rules or, for that matter, anyone else’s. A clear and cogent application of evolutionary principles entails that the products of evolved cognitive mechanisms can, in principle, take any form and have any attributes—including the attributes of being slow, deliberative, conscious, and so on. As Barrett (2015) noted, the central mantra of adaptationism is “it depends.” In other words, the functional properties one should expect to find in adaptations and by-products of adaptations will reflect the structure of the adaptive problem that mechanism evolved to solve.^16^ That is, form follows function (Williams, 1966). How could it be otherwise?

#### Holding evolutionary psychology to standards of evidence appropriate only for Fodorian modularity

The consequences of the modularity mistake are not limited to a misunderstanding of the theoretical claims evolutionary psychologists make. Confusing different levels of analysis has also led to a misapplication of standards of evidence for the resulting empirical work. As outlined above, it is possible for encapsulation and automaticity—key features of Fodor’s modules—to be intrinsic properties only if one appeals to a central agency from which modules are isolated and separated, whereas at evolutionary psychology’s functional level of analysis the central agency does not exist. However, if one fails to recognize this disconnect, then one can (mistakenly) insist that evolutionary psychology’s claims of modularity be accompanied by evidence that the proposed module is intrinsically isolated and separate. In essence, this mistake then leaves one with the notion that a module is a functional mechanism that is intrinsically isolated and separated from something else—but the “something else” is left completely unspecified.

**Fig. 6.**
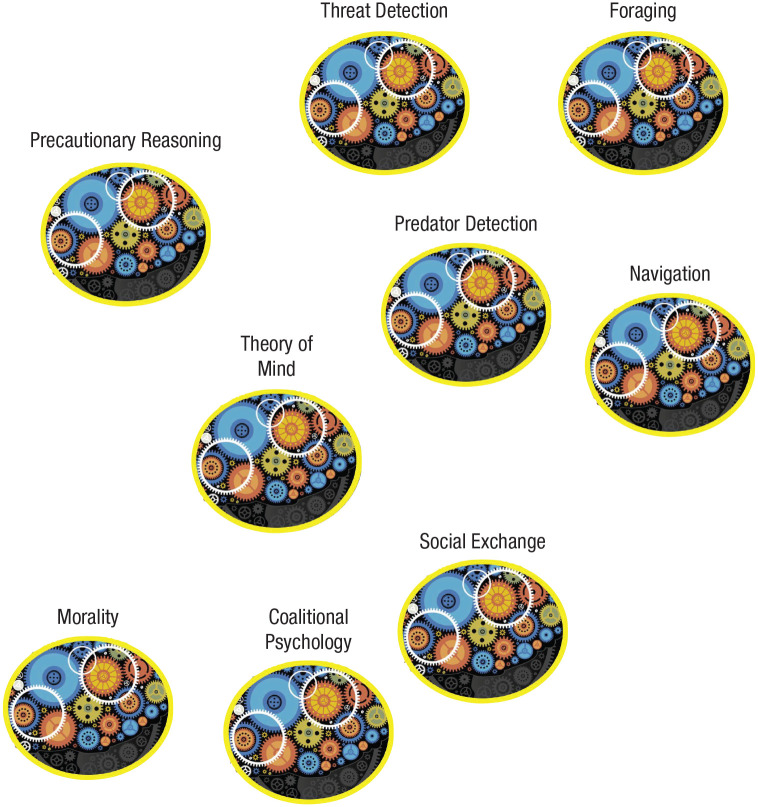
The fallacy of separate, bounded computers. The modularity mistake invites the perception that there are separate bounded computers responsible for each phenomenon studied by evolutionary psychologists (e.g., Barrett, 2016; Boyer et al., 2015; Boyer & Liénard, 2006; Cosmides et al., 2018; Ermer et al., 2006; Lopez et al., 2011; New et al., 2007).

This transposition of a standard of evidence appropriate to the intentional level but incoherent at the functional level explains a very common misunderstanding: that evolutionary psychology proposes that an entire bounded computer exists for each problem that the mind is designed to solve (see Goldfinch, 2015; Fig. 6). If one misunderstands evolutionary psychology’s claims about the mind in this way, then showing that there are cognitive processes shared among, for example, social exchange (e.g., Cosmides & Tooby, 1989, 1992, 2015) and coalitional psychology (e.g., Pietraszewski et al., 2014) or between coalitional psychology and theory of mind (e.g., Baron-Cohen et al., 1985; Ermer et al., 2006; Scholl & Leslie, 1999), then one would have seemingly invalidated the entire enterprise of evolutionary psychology (to see this view in action, see the work reviewed in Goldfinch, 2015).

Of course, this is not correct. Evolutionary psychology’s twin principles of evolvability and computational adequacy require researchers to fully describe the constellation of information-processing functions that solve each adaptive problem (Barrett, 2015; Tooby & Cosmides, 1992). However, there is no entailment that every function within that constellation has to be exclusive to solving that adaptive problem and only that adaptive problem (see also Barrett, 2012). Analogously, a constellation of processes produces a chair, and another produces a canoe. Both constellations of processes have to be described if we are to have a complete account of how chairs and canoes are made. Moreover, the two constellations of processes cannot be identical or else they would produce the same thing.^17^ However, none of this entails that the two constellations cannot share certain tools, subprocesses, or materials in common, such as hammers, saws, measuring, and so on (see also Bechtel, 2003).

**Fig. 7.**
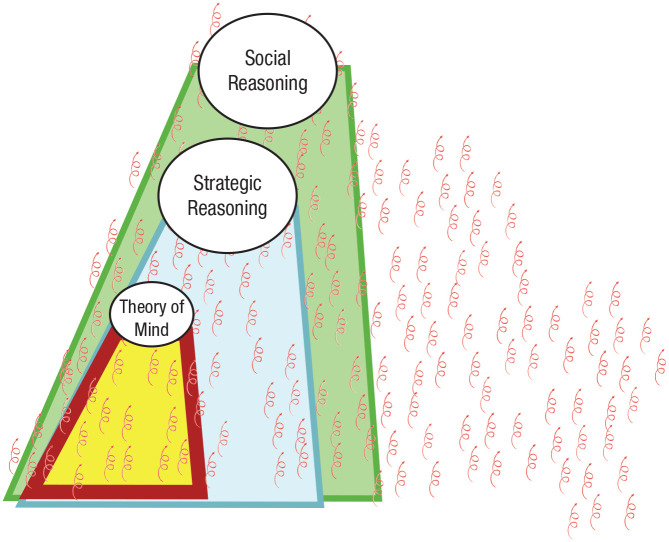
Degrees of abstraction. Because function can be described at varying degrees of abstraction, a phenomenon that describes a relatively narrower, less abstract category (such as theory of mind) will always be contained within a broader, more abstract category (such as strategic reasoning or social reasoning). Here, each squiggly line represents narrower, more specific subfunctions, and the drawn borders demarcate all of the subfunctions responsible for producing the phenomenon.

This notion of there being an entirely bounded and separate computer for each adaptive problem also explains another particularly pernicious way to falsify evolutionary psychology’s “modular” account of the mind. Functions can be described at varying degrees of abstraction, and thus so too can functionally defined mechanisms. For example, suppose you are a researcher interested in studying theory of mind. You make causal claims about the set of evolved mental functions that make possible the phenomenon of understanding others’ actions by attributing representations of underlying beliefs and desires to them. A critic armed with this bounded-computer misunderstanding of modularity can always adopt a higher degree of abstraction (see Fig. 7) by referencing a broader set functions—such as strategic reasoning or social reasoning—and then argue, “You cannot posit that this set of cognitive processes is an evolved module because you have to first show that it is not a more general process of strategic or social reasoning.” Such an argument has all of the logical merit of arguing against the claim that someone owns a kitchen sink by insisting that this cannot be proven until they have shown that the sink is not part of the kitchen.

The above misunderstandings—all born of the modularity mistake—falsely sequester evolutionary psychology’s relevance to only certain kinds of psychological processes and phenomena. They trap all parties involved into arguments that contribute little or nothing to forward progress. These misunderstandings also explain why critics believe that evolutionary psychologists are making additional and unwarranted claims about modularity that require additional evidence before these claims can be supported. Uncharitable critics can therefore portray evolutionary psychology as stubborn and narrow-minded, holding onto the obviously false idea (from their perspective) that everything is modular—which is attributed to either a failure to stay on top of “new” developments or to the blindness induced by entrenched theoretical commitments (reviewed in Goldfinch, 2015). More worryingly, communities of researchers who may otherwise be amenable to evolutionary psychology perceive themselves as being at odds with what they (mistakenly) believe to be a core claim of the field (for charitable and sophisticated critiques against modularity in evolutionary psychology, see, e.g., Bechtel, 2003; Goldfinch, 2015; Newcombe et al., 2009). In this way, the modularity mistake has not only provided facile cudgels for critics but also continues to systematically repel fellow functionalists and mechanists despite clear statements—made again and again—about what modularity means when used by evolutionary psychologists (e.g., Barrett & Kurzban, 2012; Ermer et al., 2007; Frankenhuis & Ploeger, 2007; Hagen, 2016; Tooby et al., 2005).

### Moving Beyond the Modularity Mistake

Everybody knows that something is wrong. . . . What is wrong is that not enough distinctions are being made.

—Fodor (1985, p. 1)

History is not destiny. Our science is not doomed to wallow in the modularity mistake and other similar disagreements born of confusions between levels of analysis, so long as we acknowledge that we have a problem and then do something about it. As a first step, there is much to be gained by viewing the past modularity debate through the lens of the modularity mistake. This means recognizing where differences in levels of analysis have caused unnecessary confusion and disagreement (see Box 4). Doing so will not only bring much needed clarity to a thoroughly confused literature but also bring into sharp focus that the actual scope of disagreement is much narrower than an uncorrected view suggests. Those disagreements that remain will lead to far more tractable and productive debates.

A second step will be to collectively change what we do going forward. To prevent future confusion, we suggest always marking one’s level of analysis when appealing to modularity. Fodorian modularity—or any other version of modularity that appeals to encapsulation, automaticity, and so on as intrinsic properties, or that implicitly or explicitly appeals to the existence of a central agency—should be marked as *intentional modularity*. Whereas evolutionary psychology’s notion of modularity—or any other version of modularity that either implicitly or explicitly appeals to the operation of mechanisms defined exclusively according to their functions—should be marked as *functional modularity*. Given the profound confusion created by the term “modularity,” we would suggest abandoning any unmarked use of the term going forward.

It is helpful to consider what applying this framework to modularity would look like in practice. Let us return to the researcher who is interested in the broad phenomenon of theory of mind. This researcher could at one level meaningfully ask what intentional-level attributes different aspects of this phenomenon have. For example, the researcher might discover that ascribing mental states to agents by observing their actions is automatic, effortless, intuitive, unconscious, and not “penetrable” to one’s insight or reasoning. These are perfectly coherent descriptions of the mind’s information-processing at an intentional level of analysis. Such attributes can then be used to argue that the processes that “you” use to ascribe mental states to others because of the actions they perform constitutes an *intentional module*. A research program and meaningful debate about the intentional modularity of theory of mind would then center around which aspects of theory of mind have which particular intentional-level attributes (e.g., Apperly et al., 2006).

In contrast, at a functional level of analysis, the researcher may additionally propose that the ability to ascribe mental states to agents by observing their actions is the result of a *functional module*. Such a statement would imply that the researcher will, in step-by-step mechanistic detail, establish how this functional module works. For example, that the input cue [agent approaches object] results in the output of representations of a desire for the approached object and a true belief about its location (Wertz & German, 2013). From there, additional work would be required to establish (a) what precise class of distal inputs trigger the mental entities [agent], [object], and [approach], (b) what mechanistic consequences this representational output of [desire + true belief] has within the cognitive architecture and eventually on an agent’s behavior in the distal world (see Block, 1998; Dennett, 1969/2002, 1995), and so on. At every step in this process, the researcher is exclusively appealing to the operation of mechanisms and their attributes (i.e., their input/processes/output logic). At no point does a central agency enter the picture. Moreover, using the term “functional module” does not entail claiming that the processes involved are automatic, effortless, intuitive, unconscious, and so on. Instead, the characteristics of the processes within each functional module will be dictated by the structure of the problem it solves.

In the case of evolutionary psychology, we would go one step further and suggest that researchers abandon the use of the term “modularity” altogether—at least for the foreseeable future. The confusions outlined above are more than enough justification for such a proscription.^18^ Evolutionary psychologists would be better served by referring to the entities that they study as functional mechanisms or functional systems, which creates as much distance as possible from modularity and its confusions. In any case, we believe the language used within the functional level of analysis is less important than clearly marking the level of analysis at which one is operating—not just for evolutionary psychologists, but for everyone.

In this post-modularity-mistake world, the theoretical tenets of evolutionary psychology are not altered. The central issues of an adaptationist analysis—determining whether the psychology that is being proposed satisfies evolvability criteria and is computationally adequate—not only will remain but will be brought into sharper relief because there will be fewer incidental debates in the way. Fodorian modularity, in turn, retains the valuable descriptions of psychological processes from a first-person, intentional-level perspective.

### Conclusion

What has seemed to be an important but interminable debate about the nature of (massive) modularity is better conceptualized as the modularity mistake. Clarifying the level of analysis at which one is operating will not only resolve the debate but also render it moot. In its stead, researchers will be free to pursue much simpler, clearer, and more profound questions about how the mind works. If we proceed as usual, we will end up back in the same confused place where we started in another 40 years—arguing once again about who’s on first.

Confusing or collapsing across different levels of analysis is not just a problem for modularity and evolutionary psychology. Rather, it is the greatest problem facing early-21st-century psychology, dwarfing even the current replication crisis. Since at least the days of the neobehaviorists (e.g., Tolman, 1964), the ontology of the intentional level has become mingled with the functional level in all areas of the cognitive sciences (see Stich, 1986). Constructs such as thinking, reasoning, effort, intuition, deliberation, automaticity, and consciousness have become misunderstood and misused as functional-level descriptions of how the mind works. Appeals to a central agency who uses “their” memory, attention, reasoning, and so on have become commonplace and unremarkable. Even the concept of cognition itself has fallen into the same levels-of-analysis confusion seen in the modularity mistake.^19^ In the process, a shared notion of what it means to provide a coherent functional level (or mechanistic) description of the mind has been lost.

We do not bring up these broader issues to resolve them here. Rather, we wish to emphasize what is at stake when it comes to being clear about levels of analysis. If we do not respect the distinctions between levels, no amount of hard work or mountains of data that we will ever collect will resolve the problems created by conflating them. The only question is whether we are willing to begin the slow, difficult—but ultimately clarifying and redeeming—process of unconfounding the intentional and functional levels of analysis. The modularity mistake is as good a place as any to start.

## Transparency

*Action Editor:* Laura A. King

*Editor:* Laura A. King

## Notes

1. Marr’s computational level describes the problem something is solving, whereas the lower, algorithmic/representational level describes how that problem is solved. Both levels fall within the purview of the functional level outlined here, as both entail rules of abstract causation carried out exclusively by mechanisms.

2. We use the term “functional” as opposed to “computational” out of an interest in being able to refer to the multiple functions that exist within organisms rather than just describing the particular function of computation. That said, for the purposes here, the reader may readily substitute “computational” for “functional,” so long as the reader interprets the former as a token of function rather than a modeling method (i.e., computational models are something else entirely). Moreover, we are not describing here the more precise notion of functionalism as a philosophical doctrine applied to the mind (e.g., Block, 1978), particularly because we believe this doctrine has itself been a victim of confusion between levels of analysis (e.g., Fodor, 1983, p. 25)—this is another confusion that will also have to be addressed (for trenchant analyses that match our position, see, e.g., Block, 1998; Dennett, 1969/2002, 1995; Tooby & Cosmides, 1990).

3. We suspect that some readers will find this claim—that Fodorian modularity exists at the intentional level of analysis—tendentious, but they should not. Fodor was consistent throughout his work (e.g., Fodor, 1968, 1976, 1985, 1987, 1998) in defending a causal science of belief/desire psychology—what Dennett (1991b) called his “intentional realism.” In other words, Fodor was an avowed advocate of the two-level-of-analysis worldview (Fodor, 1975, 1976). That is, and as we explain later, Fodor collapsed intentional and functional levels together. We take this to be a consequence of his being (a) on the ground floor, as it were, of functionalism à la Putnam; (b) an inheritor of the broader mind/body issue of philosophy, in which all but the distinction between mind and matter was triaged away, reasonably enough; and (c) an analytic philosopher reacting against Carnap’s reductionism (see also Dennett, 1995). Leaving aside the issue of collapsing or confusing the intentional and functional levels together (which is a category mistake), we wish to be clear that we do not mean that the intentional level of analysis is unscientific. Indeed, we believe that intentional-level descriptions need to be a part of a complete scientific description of the mind; the problem is when they are confused with or stand in for functional-level descriptions.

4. In fact, the relatively few references to modularity in these early articles nearly always included extensive paraphrasing that appealed to functional specialization. That is, this early work went out of its way to try to prevent the very misunderstanding that indeed happened. Here is a representative passage: “Our view, then, is that humans have a faculty of social cognition, consisting of a rich collection of dedicated, functionally specialized modules (i.e., functionally isolable subunits, mechanisms, mental organs, etc.)” (Cosmides & Tooby, 1992, p. 163).

5. Fodor’s treatment of Gall in *Modularity of Mind* is patchy. Early in the book (e.g., pp. 20–22, 37) he seems to mischaracterize Gall’s position by treating vertical faculties as independent from, rather than orthogonal to, horizontal faculties, but then corrects himself later on (e.g., p. 72). We do not go into this further here aside from suggesting that the curious reader look into the original Gall quotations Fodor used from Hollander (1920). From our reading, Gall seems to have been actively distinguishing between intentional and functional levels of analysis and may have been one of the first to make the distinction clearly with respect to the mind (e.g., Hollander, 1920, pp. 213, 243–245; see, however, Aristotle, 350 B.C.E./1994).

6. Fodor’s explicit goal was to argue against “New Look” theorists (such as Bruner) who were arguing that beliefs, desires, goals, and so on can permeate even the earliest stages of perception (p. 73). He was sympathetic to their goal—which at the time was to argue against the behaviorists’ perception-as-reflex account (which Fodor also disagreed with). Fodor simply thought they reacted too far in the other direction (pp. 82–83). Fodor is also commonly misunderstood as arguing that perception itself is encapsulated, but he certainly did not: “Input analysis may be informationally-encapsulated, but perception surely is not” (p. 73).

7. Moreover, it is illuminating to consider what would be required for the Shepard visual illusion to never occur. That is, what would it look like for processes in the visual system to be no longer considered encapsulated and therefore no longer modular? At a functional level, this would require the existence of an additional mechanism in the mind whose function is to shuttle information from the mechanisms responsible for handling linguistically communicated declarative knowledge (i.e., the textbook explanation that the two monsters are in fact the same size) to the mechanisms responsible for producing the first-person experience of the apparent size difference (see also Barrett, 2015; Rozin, 1976). If such a mechanism did indeed exist, then at the intentional level of analysis, “you” (i.e., the central agency) would be able to influence visual perception, and the visual system would cease to meet Fodor’s criterion of encapsulation. That is, by adding a mechanism at the functional level of analysis, we have lost a module at the intentional level.

8. That is, there is no way aside from the banal point that causation has boundedness (i.e., such that functions are separable). In this sense, every single mechanistic processing step within the mind is equally encapsulated: What a mechanism does, it does somewhere, which is different from all of the other stuff happening everywhere else.

9. All mechanisms at the functional level of analysis are fully “automatic”; there cannot be an outside agency to step in and intervene. This is true even when the mechanism does something that one would think of as being deliberative, effortful, or flexible at the intentional level (see Braitenberg, 1984; Carruthers, 2003).

10. That Fodor’s notion of modularity ran counter to the notion that “you” are in complete control of your mind may help explain why even the attributes within his framework that could plausibly exist at a functional level of analysis seem to presented at an intentional level. Even for domain specificity, for example, Fodor essentially argued that something is domain-specific to the degree that its inputs seem counterintuitively specific or if it ignores something that seems intuitively relevant (e.g., Fodor, 1983, pp. 49–50; Fodor, 2000, pp. 60–63). In other words, something is domain-specific if it behaves in a way that “you” would not (see also Boyer & Barrett, 2016; Hagen, 2016).

11. Evolutionary psychology also occasionally approaches the mind at the intentional level of analysis, particularly when different phenomenological states produce different consequences out in the world. For example, if something in the mind is experienced as automatic or unconscious it cannot be explicitly communicated to others (e.g., Kurzban, 2010; Kurzban & Athena Aktipis, 2007; Von Hippel & Trivers, 2011).

12. “Adaptation” here includes phenotypic design that is the product of gene-culture coevolution (e.g., Barrett, 2015; Boyd & Richerson, 1985; Wertz & Moya, 2019).

13. In fact, the authors of early work in evolutionary psychology seem to have anticipated that there might be some confusion between what they meant and what Fodor meant by modularity, as they typically went out of their way to clarify what they meant by modularity whenever the term was used. For example, the relatively few references to modularity in the foundational edited volume, *The Adapted Mind* (1992), nearly always included paraphrasing that referenced functional specialization (i.e., functional individuation; for an acknowledgment of this as a different meaning of modularity than his own, see Fodor, 2000, Chapter 4, Footnote 3).

14. Confusing the entire functional level of analysis for the “module” slot within the intentional level of analysis (see Fig. 2) also explains why clarifications of evolutionary psychology’s meaning of modularity as functional specialization elicits counterresponses involving System 1/System 2 dichotomies (e.g., Chiappe & Gardner, 2012)—which are of course thinly veiled redescriptions of the intentional level’s me/not-me distinction. That is, this confusion explains the otherwise mysterious happenstance that dual-systems theory and Fodorian modularity seem to regularly co-occur in the literature (see Barrett, 2015, p. 266). We would note that dual-systems theories of cognition, which historically used an intentional-level description of the mind as a stand-in for a functional level description, are, to their credit, now going through the growing pains of unconfounding the two and fleshing out a functional-level description (e.g., Evans & Stanovich, 2013). The same confusion also explains the otherwise gasp-inducing claim made by Fodor (1983, 2000) that functionalism (or computationalism) could only ever be applied to noncentral systems (i.e., modules), whereas central systems must remain forever a mystery. Fodor’s claim was not just an aesthetic preference (cf. Murphy, 2019); it follows logically from Fodor’s intentional-level-of-analysis worldview (see Fig. 2).

15. We refer to this practice as *binning*—that is, assigning a phenomenon to one option within an either/or categorical distinction. Binning is a widespread practice; other examples include identifying aspects of cognition as rational versus irrational, fast versus slow, and so on. Barrett (2015) called these kinds of either/or dichotomies “dualist” distinctions, and Minsky (2006) called them “dumbbell” theories. Not all instances of binning on the basis of dualist distinctions are incoherent, but the practice is overused and often comes at the expense of providing causal explanations for mental processes and invites conceptual errors of the type described here.

16. Certain properties will also reflect constraints and other non-hill-climbing, non-anti-entropic evolutionary processes, such as drift (See Dennett, 1995; Smith & Winterhalder, 1992; Tooby et al., 2003).

17. This is a different point than the type/token distinction (e.g., Barrett, 2007)—that the exact same process can produce multiple instances (tokens) of the same type. For example, one need not posit a different set of tools for each identical canoe made.

18. There is another reason to abandon the term: There is an older, more precise meaning of modularity that predates Fodor; has already been in use in other fields such as physiology, developmental and evolutionary biology, artificial intelligence, and software engineering; and is not equivalent to evolutionary psychology’s notion of functional specialization. This original version of modularity is a characteristic of a system—described at either a functional or implementation level—in which the system executes its function by decomposing the problem it solves into hierarchically arranged tasks and subtasks (Ehrenfeld et al., 2013). Hierarchically arranged systems nested within other systems solve these types of problems in which “no ‘demon’ or other local process can know enough about the overall situation to make good decision; but no top-level manager can know enough details either” (Minsky, 1974/1975, p. 60; see also Lorenz, 1948/1996). In this context, the term “module” is a derivative of what Simon called the property that emerges from such hierarchical systems, “near decomposability” (e.g., Simon, 1969/1996, p. 204). “Nearly decomposable units, arranged hierarchically,” does not exactly roll off the tongue. So this longer expression is shortened to “modules”: “Many complex systems found in nature or that humans have designed are organized hierarchically from components—modules—that have some degree of independence. Herbert Simon called such systems ‘nearly decomposable’” (Barto et al., 2013, p. 13). That is, modules in this sense are clusters of higher within versus between causal interaction, arranged hierarchically (that *do* interact, by design)—and are meaningful at either an implementation or functional level of analysis. See also Baldassarre and Mirolli (2013), Barrett (2012), Needham (1933), Schlosser and Wagner (2004), and Winther (2001).

19. Cognition can be understood as a class of mental process at an intentional level (e.g., referring to agents’ thinking as opposed to their feelings and motivations). Or it can be understood as a description of the mind at a functional level of analysis, at which it refers to everything that the mind does (e.g., “hot,” “nonthinking” processes such as emotions). We believe that the stalling of the cognitive revolution itself (Núñez et al., 2019) can be traced back to a confusion between these two different senses of the concept.
